# Advanced mechanical circulatory support for post-cardiotomy cardiogenic shock: a 20-year outcome analysis in a non-transplant unit

**DOI:** 10.1186/s13019-016-0430-2

**Published:** 2016-02-18

**Authors:** Maziar Khorsandi, Kasra Shaikhrezai, Sai Prasad, Renzo Pessotto, William Walker, Geoffrey Berg, Vipin Zamvar

**Affiliations:** Department of Cardio-Thoracic Surgery, Royal Infirmary of Edinburgh, Edinburgh, UK; Department of Cardio-Thoracic Surgery, Golden Jubilee National Hospital, Glasgow, UK

**Keywords:** Extracorporeal circulation, Heart-assist devices, Post-cardiotomy, Shock

## Abstract

**Background:**

Post-cardiotomy cardiogenic shock (PCCS) has an incidence of 2–6 % after routine adult cardiac surgery. 0.5–1.5 % are refractory to inotropic and intra-aortic balloon pump (IABP) support. Advanced mechanical circulatory support (AMCS) can be used to salvage carefully selected number of such patients. High costs and major complication rates have lead to centralization and limited funding for such devices in the UK. We have looked the outcomes of such devices in a non-transplant, intermediate-size adult cardiothoracic surgery unit.

**Methods:**

Inclusion criteria included any adult patient who had received salvage veno-arterial extra-corporeal membrane oxygenation (V-A ECMO) or a ventricular assist device (VAD) for PCCS refractory to IABP and inotropic support following cardiac surgery from April 1995-April 2015.

**Results:**

Sixteen patients met the inclusion criteria. Age range was 34–83 years (median 71). There was a male predominance of 12 (75 %). Overall, 15 (94 %) had received ECMO of which, 10 (67 %) had received central ECMO and 5 (33 %) had received peripheral ECMO. One patient (6 %) had a VAD. The most common complication was haemorrhage. Stroke, femoral artery pseudo-aneurysm, sepsis and renal failure also occurred. Thirty-day survival was 37.5 %. Survival rate to hospital discharge was 31.2 %. All survivors had NYHA class I-II at 24 months follow-up.

**Conclusions:**

Our survival rate is similar to that reported in several previous studies. However, the use of AMCS for refractory PCCS is associated with serious complications. The survivors in our cohort appear to maintain an acceptable quality of life.

## Background

Post-cardiotomy cardiogenic shock (PCCS) occurs in 2–6 % of patients undergoing surgical revascularization or valvular surgery [1–4]. Approximately 0.5–1.5 % of patients are refractory to maximal inotropic and intra-aortic balloon pump (IABP) support [5]. Post-cardiotomy cardiogenic shock occurs in perioperative cardiac surgery in patients with normal preoperative myocardial function as well as those with pre-existing impaired function [6]. Refractory PCCS leads rapidly to multi-organ dysfunction and is nearly always fatal [4, 7–9] without the use of advanced mechanical circulatory support (AMCS). AMCS devices such as extracorporeal membrane oxygenation (ECMO) and ventricular assist devices (VAD) have been used to salvage patients who develop refractory PCCS. Survival to hospital discharge is variable [1–3, 5, 10–12] though long term survival and reasonable functional outcome can be achieved [11, 13–15]. However, these devices are associated with serious complications [1, 2, 11, 16–18] and are costly [9, 19, 20]. The UK National Health Service’s (NHS) proposal to centralise AMCS funding to a few, larger cardiothoracic units has been controversial [15]. Some argue that such a move would remove this potentially, life-saving resource from those cardiothoracic surgery departments excluded from AMCS funding [15]. This prompted us to assess the outcome of the use of AMCS in a non-transplant, intermediate-sized unit (annual workload approximately 900 to 1000 major cardiac surgical operations) in Edinburgh, UK.

## Methods

We retrospectively assessed our experience with AMCS in refractory PCCS over a 20-year period. These data were collected prospectively by the Royal Infirmary of Edinburgh cardiac surgery database. Our inclusion criteria included any adult patient from April 1995 to April 2015 who had received salvage V-A ECMO or VAD for PCCS refractory to IABP and maximal inotropic support following adult cardiac surgery. We gained information regarding the patients’ follow-up status from accessing the cardiology follow-up clinic letters on the TrakCare^R^ system, which captures in-patient and out-patient clinical data.

The AMCS devices utilised by our unit in duration of the study included: The Levitronix^R^ CentriMag II for ECMO and Medtronic Bio-Medicus^R^ 560 for VAD support.

## Results

There were 18 patients who met the inclusion criteria. We excluded two patients who had had AMCS for refractory PCCS. One patient was in the paediatric age group and had been operated on prior to relocation of paediatric cardiac surgery services to the Royal Hospital for Sick Children, Glasgow in year 2000. Another patient was excluded due to the lack of recorded clarity in the database on the type of AMCS support used, any potential complications and the short and the long-term outcome of this individual. In the remaining 16 patients the age range was 34–83 years (Median 71 years).

**Fig. 1:**
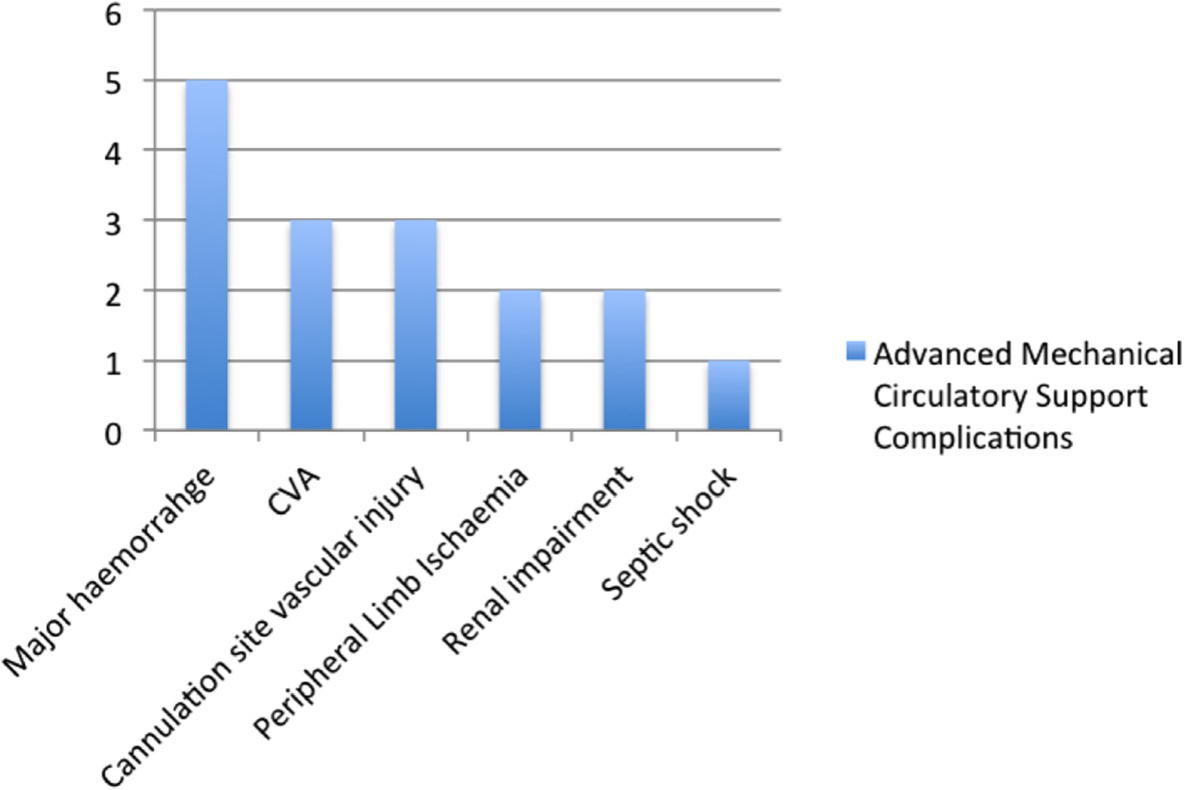
Illustrates the number of complications in our cohort

Among the remaining 16 (89 %) patients (Table. 1), there was a large male predominance of 12 (75 %). Five patients (31.25 %) had undergone re-operative cardiac surgery. One patient (6.25 %) had undergone AMCS following the repair of an ascending aortic transection after a road traffic accident. Overall, 15 patients (94 %) had received a single run of V-A ECMO of which number, 10 (67 %) had received central ECMO and 5 (33 %) had received peripheral ECMO. One patient (6 %) had VAD. The mean duration of AMCS was approximately 5.4 days (Range < 1 day – 33 days). The most common procedure-related complication was major haemorrhage. The incidence of major cerebrovascular accident, peripheral limb ischaemia, femoral artery pseudo-aneurysm, septic shock and renal failure requiring renal replacement therapy was 18.75 %, 12.5 %, 6.25 %, 12.5 % and 18.75 % respectively (Fig. 1). Logistic EuroSCORE ranged from 2.08 to 73.26 (mean 20.21, SD = 17).

**Table 1:** Illustrates patient characteristics, the type of mechanical circulatory support and outcomes over the 11-year study period

	Age & gender	Co-morbidities & logistic euroSCORE	Date of surgery	Original operation	Duration and mode of AMCS	AMCS complication/s	Outcome
Patient 1	76 year old male	MI	2012	Re-do sternotomy and AVR	Salvage peripheral VA ECMO due to postoperative pulmonary haemorrhage	Femoral artery cannulation site pseudoaneurysm	Alive
		CABG					
		Moderate LVSD				Major haemorrhage from cannulation site	NYHA I (No breathlessness of exertion, back to work)
		Hypertension					
		Hypercholestrolaemia					
		ICD for VF					
		CVA					
		EuroSCORE = 27.48					
Patient 2	40 year old male	MV repair	2014	Re-do, Re-do sternotomy for type A aortic dissection: Bentall procedure.	Salvage RVAD due to VF arrest and asystolic LV after CPB	Major haemorrhage and re-exploration in the operating theatre	Alive
		MVR					
							NYHA II (Breathless on exertion)
		Moderate LVSD					
		Marfan’s syndrome					
		AF					
		LogEuroSCORE = 29.18					
Patient 3	82 year old male	Severe LVSD	2006	MV Repair and CABG	3 Days	Could not be weaned from ECMO with severe biVent failure	Died in CTICU
		MI			VA ECMO as unable to wean from CPB		COD: BiVent failure
		Severe TVD					
		LogEuroSCORE = 17.45					
Patient 4	72 year old Female	Good LV function	2011	AVR	9 Days	Septic shock	Died in CTICU
		Moderate MR			VA ECMO as unable to come off CPB	Peripheral ischaemia	COD: Septic shock
		EuroSCORE = 12.11					
Patient 5	71 year old male	Urgent/Emergency Surgery	2011	CABGx3 and AVR	2 Days	ECMO cannulation site bleeding and haematoma explored	Died in CTICU
					Peripheral VA ECMO as unable to come off CPB	Renal failure^a^	COD: Shock (unknown cause)
		MI (< 90 days)					
		Severe LVSD					
		Severe TVD					
		Anaemia					
		LogEuroSCORE = 26.35					
Patient 6	83 year old female	Urgent/Emergency Surgery	2012	MVR and CABG ×1	< 1 Day	None	Died in CTICU
					Peripheral VA ECMO as unable to come off bypass and awaited family to see patient last time		COD: BiVent failure
		MI (< 90 days)					
		Severe LVSD					
		Acute severe MR					
		Cardiogenic shock					
		LogEuroSCORE = 73.26					
Patient 7	70 year old male	MI	2013	Re-do sternotomy and AVR	33 Days	Major CVA	Died in HDU
		CABG			VA ECMO. Successfully weaned from ECMO		COD: severe Respiratory failure
		CVA					
		Hypertension					
		Hypercholestrolaemia					
		Good LV					
		LogEuroSCORE = 14.31					
Patient 8	72 year old male	Moderate LVSD	2013	Re-do sternotomy and AVR	< 1 Day	ECMO cannulation femoral artery dissection	Died in CTICU
		CABG					
		Atrial flutter			VA ECMO after iatrogenic aortic dissection during Femoral cannulation for bypass	Major haemorrhage	COD: Major CVA
		Hypertension					
		LogEuroSCORE = 13.09				Major CVA	
Patient 9	51 year old male	Moderate LVSD	2013	Re-suspension of Aortic valve and repair of type A aortic dissection	1 Day	Major cannulation site haemorrhage	Died in CTICU
		LogEuroSCORE = 13.13			Peripheral VA ECMO		COD: Haemorrahgic shock and BiVent failure
Patient 10	34 year old female	Good LV	2014	IVC Leiomyosarcoma resection	3 Days	None	Died in CTICU
		LogEuroSCORE = 2.08			VA ECMO		COD: BiVent failure from acute MI
Patient 11	65 year old male	Urgent surgery	2013	CABG	2 Days	Renal failure^a^	Died in CTICU
		ACS (Unstable angina)			Salvage VA ECMO	Hepatic failure	COD: MODS
		LogEuroSCORE = 4.55				Pulmonary oedema	
Patient 12	71 year old male	Peripheral vasculopath	2015	CABG	3 Days	Major haemorrhage: Re-opening for bleeding ×4	Died in CTICU
		LogEuroSCORE = 3.67					
					VA ECMO as unable to wean from CPB	Peripheral leg ischaemia	COD: biventricular failure and septic shock
Patient 13	49 year old male	Emergency Surgery	1997	CABG	VA ECMO	Note recorded	Alive (Died 2004) NYHA II
		PVD					
		Intra-operative MI					
		LogEuroSCORE = 5.69					
Patient 14	69 year old male	Active IE	2004	MVR and CABG for mitral valve IE	VA ECMO	CVA and seizures Renal failure^a^	Alive NYHA II
		Emergency surgery					
		Moderate LVSD					
		LogEuroSCORE = 21.73					
Patient 15	41 year old female	Emergency surgery	2005	Aortic transection and diaphragmatic rupture	VA ECMO	Not recorded	Alive NYHA I
		Good LV					
		LogEuroSCORE = 25.50					
Patient 16	59 year old male	MI (< 90 days)	2006	Type A aortic dissection	2 Days	Not recorded	Died COD: Bivent failure
		Severe LVSD			Peripheral VA ECMO as unable to come off bypass		
		Emergency surgery					
		LogEuroSCORE = 33.90					

Abbreviations: *ACS* acute coronary syndrome, *AF* atrial fibrillation, *AMCS* advanced mechanical circulatory support, *AVR* aortic valve replacement, *CABG* coronary artery bypass grafting surgery, *CPB* cardiopulmonary bypass, *COD* cause of death, *BiVent failure* biventricular failure, *MVR* mitral valve replacement, *IE* infective endocarditis, *CVA* cerebrovascular accident, *IVC*, inferior vena-cava, *NYHA* New York Heart Association, *CTICU* cardiothoracic intensive care unit, *HDU* high dependency unit, implantable cardioverter defibrillator, *MI* myocardial infarction, *LVSD* left ventricular systolic dysfunction, *TVD* triple vessel coronary artery disease, *LV* left ventricular, *MR* mitral regurgitation, *PVD* peripheral vascular disease, *MODS* multi-organ dysfunction syndrome, *VF* ventricular fibrillation, *VAD* ventricular assist device, *VA* veno-arterial;

^a^All patients with renal failure required renal replacement therapy

**Fig. 2:**
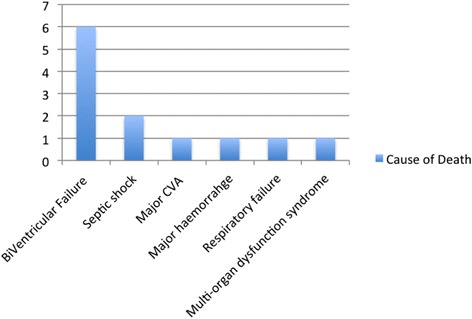
Illustrates the cause of death in our cohort

**Fig. 3:**
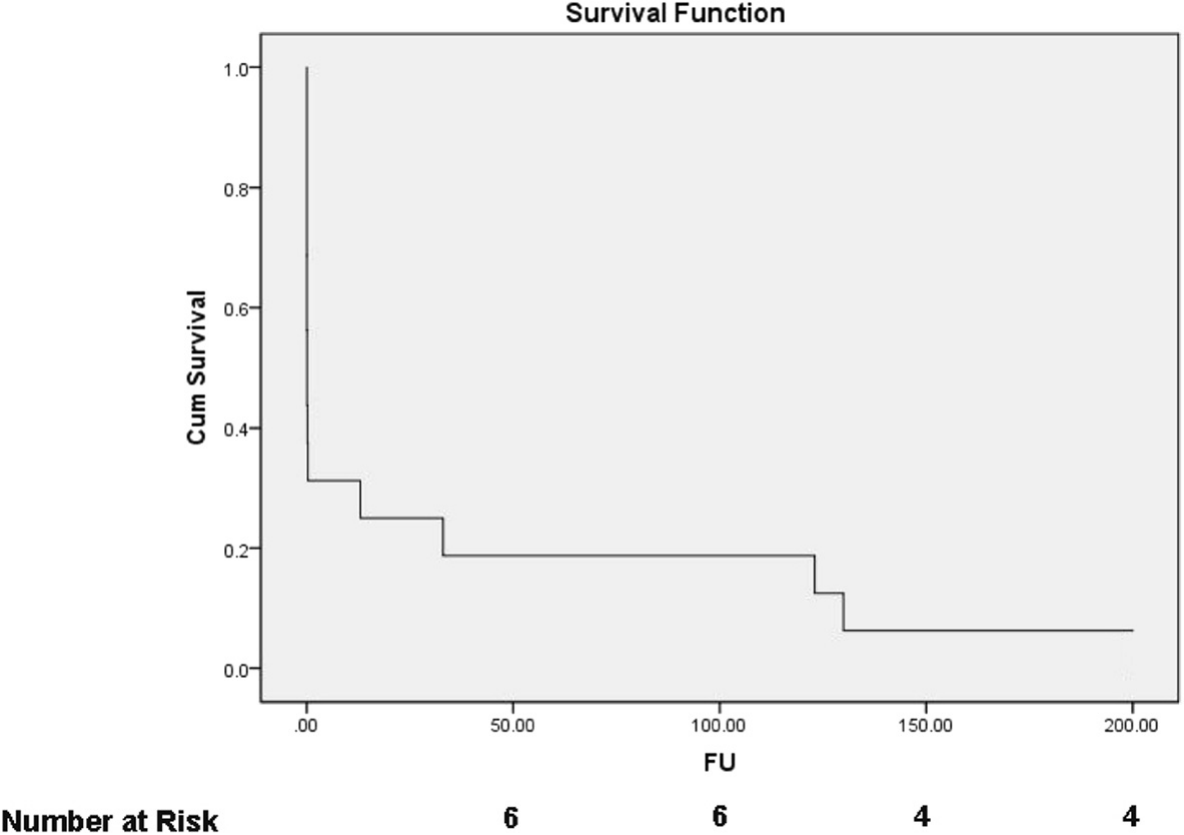
Kaplan-Meier curve of survival. FU: follow up (months), Cum Survival: cumulative survival

Most common cause of death (COD) was refractory biventricular failure (37.5 %, Fig. 2), in which group of patients, AMCS was withdrawn. One patient died due to combination of biventricular failure and haemorrhagic shock whilst on VA ECMO. Thirty-day survival was 37.5 % (Fig. 3). Our survival rate to hospital discharge was 31.2 %. Upon reviewing the consultant cardiologist follow-up clinic letters on the TrakCare^R^ database, all survivors who were discharged home were alive at 24 months and had NYHA class I-II functional status on follow-up.

Advanced age, the emergent nature of surgery, pre-existing, preoperative severe left ventricular impairment were identified as possible factors leading to an adverse outcome. These findings however are limited due to the small number of subjects and the retrospective nature of the study.

## Discussion & review of the literature

AMCS have been in evolution for almost 50 years. AMCS devices developed in parallel with the cardiopulmonary bypass (CPB) machine by John Gibbon in 1953 [21, 22]. Soon after its inception, the CPB machine was being used to salvage patients following “failed” cardiac surgery. In 1966, Michael DeBakey used the first LVAD to support a patient who was in refractory PCCS [21]. After the first heart transplantation by Christiaan Barnard in 1967 and widespread acceptance of transplantation, AMCS devices have been regularly used to sustain those patients in refractory PCCS to allow time for decision for further management with implantable devices or heart transplantation [21].

There are two forms of ECMO; veno-venous (VV) which is usually utilized to support isolated respiratory failure and veno-arterial (VA) ECMO used to support cardiac, respiratory or mixed cardiorespiratory failure. Both types of ECMO can be applied centrally and peripherally [23, 24]. There are two types of VADs. Short-term, percutaneous VADs, which can be inserted in the catheter laboratory, serve as a temporizing measure to stabilize patients in acute cardiogenic shock. More long-term, surgically-implanted VADs are designed to increase patient’s life span and/or bridge patients to transplantation [15]. VADs can be used to support the failing right ventricle, left ventricle or both. According to the UK VAD registry, scarcity of donor hearts has stimulated consideration of implantable AMCS devices for more prolonged circulatory support [20]. Recent studies have demonstrated that modern continuous flow AMCS devices such as CentriMag^R^ pumps with magnetically levitated rotors and more effective modern oxygenators, can be more easily implanted, leading to much better survival in patients with post-cardiotomy cardiogenic shock in the recent years [25–27].

In our literature search we identified 15 major articles. In the largest cohort, Hernandez et al. [3] collated data from 5,735 patients who underwent salvage VAD support for refractory PCCS. They reported 54.1 % survival rate to hospital discharge. They concluded that VAD is a valuable, life-saving therapeutic manoeuvre. Rastan et al. [5] performed ECMO support in 516 patients with refractory PCCS. A total of 24.8 % of patients survived to discharge to the community. However, after 5 years 13.7 % were alive and 17.4 % of patients suffered severe morbidity [5]. In another large cohort of 219 patients Doll et al. [28] reported 24.8 % survival to discharge and 16.9 % 5-year survival for their cohort of refractory PCCS patients who had received salvage ECMO support. Hsu et al [12] reported a cohort of 51 patients who had undergone cardiac surgery and suffered refractory PCCS requiring ECMO circulatory support. The 30-day, 3-month and 1-year mortality rates were 49, 65 and 71 % respectively. Mehta et al. [13] reported a large cohort of 1,279 patients who had undergone VAD for circulatory support for refractory PCCS. They claimed that 584 (45.7 %) patients were successfully weaned from VAD and 323 (25.3 %) patients survived to discharge from the hospital. In a small cohort of 12 patients with refractory PCCS, DeRose et al. [2] reported that 9 patients (75 %) survived to discharge from the hospital with LVAD implantation, 8 (67 %) survived to transplantation and 1 (8 %) successfully underwent explanation of LVAD not requiring transplantation. However, they reported 42 % rate of LVAD related infective complications.

With respect to longer term outcomes; a study by Ko et al. [11] reported a cohort of 76 patients undergoing ECMO support for refractory PCCS. They reported that, although 46 patients (60.5 %) were successfully weaned from ECMO, 20 (26.3 %) survived to discharge. However all survivors were reported to be of New York Heart Association (NYHA) I and II functional status on 32 +/- 22 month follow-up. This quality of life finding is in keeping with the findings in our cohort. Pennington et al. [17] reported refractory PCCS support with VAD with 37 % survival to hospital discharge. They reported that all survivors were “leading active lives”. In 72.7 % of survivors ejection fraction had normalized on follow-up echocardiogram. AMCS devices also have disadvantages. They are currently not funded within the cardiac surgery tariff and are quite costly [9, 19, 20]. According to a study conducted at the University Hospital of South Manchester [9] (an NHS institution), the cost of capital equipment, device maintenance and single-use elements (e.g. tubing) of the CentriMag^R^ device is £3542. The total per patient cost of VAD and ECMO were reported as £15 669 and £8616 respectively. However the costs associated with the device and the ICU stay may vary substantially depending on the duration of AMCS. The cost of implantable VAD per patient receiving a device is £173,841 or US$316,078. The most costly aspect is implantation of the device (£63,830, US$116,056) and the initial hospital stay in the ITU and ward totals as much as £14,500 or US$26,364 [29]. A clinical trial indicated the cost of quality adjusted life year for ECMO in the UK to be £19,252 [30]. Also there are reports [1, 2, 11, 16–18] of serious device-related complications including: bleeding, thrombus formation and embolization, cerebrovascular accidents, device infection, limb ischaemia and multi-organ dysfunction syndrome/failure. However, there is evidence that on-the-table, early implantation of AMCS devices, prior to leaving the operating theatre from the initial operation, substantially improves survival as compared to late implantation out-with the operating theatre [6, 26]. Given the reported survival rates and the extent of resources involved, we recommend that each case should be assessed in its own individual merit. Involvement of a cardiac surgeon, other than the surgeon involved in the initial operation, the clinical director of the department of cardiac surgery, as well as involving the perfusionist and the on-call anaesthetist in the decision making process is imperative in gaining optimal outcomes for patients undergoing AMCS for refractory PCCS while optimising the cost-benefit equation.

## Conclusion

AMCS devices can be used to salvage some patients with refractory PCCS who would otherwise have not survived. However, ACMS are associated with high rates of severe, systemic and device-related complications as well being costly. We recommend team approach to decision-making and early application of AMCS to the few carefully selected patients with refractory PCCS in order to optimise the cost-benefit equation. Our results reflect findings from previous studies. Our study showed survivors enjoyed a reasonable quality of life.
